# Quality of Life and Surgical Outcome of Transoral Endoscopic Thyroidectomy Vestibular Approach (TOETVA) versus Open Thyroid Surgery: Experience from a Single Center in Vietnam

**DOI:** 10.1155/2022/2381063

**Published:** 2022-10-11

**Authors:** Hau Xuan Nguyen, Hien Xuan Nguyen, Hoai Thi Hoang, Quang Van Le

**Affiliations:** ^1^Department of Oncology, Hanoi Medical University, No. 1 Ton That Tung Street, Dong Da District, Hanoi, Vietnam; ^2^Department of Oncology and Palliative Care, Hanoi Medical University Hospital, No. 1 Ton That Tung Street, Dong Da District, Hanoi, Vietnam

## Abstract

**Background:**

It has been widely assumed that TOETVA has demonstrated a new technique and a promising approach as it is both minimally invasive and optimally cosmetic. The objective of this study was to assess the surgical outcome, aesthetic satisfaction, and postoperative quality of life of TOETVA in comparison with open thyroid surgery. *Patients and Methods*. The study was designed as a prospective study, in which 121 patients from a single center in Vietnam underwent thyroid surgery, and the study was divided into two groups: 60 patients in the TOETVA group and 61 patients in the open surgery group. The patients have been followed up including surgical outcomes, cosmetic satisfaction, and quality of life. These criteria were assessed at 4 weeks, 8 weeks, and 12 weeks after the surgery using SF-36 and thyroid surgery-specific questionnaire.

**Results:**

Patients in the TOETVA group are significantly younger than patients in the open surgery group (35.8 + 10.3 vs 46.9 + 11.5, *p* < 0.001). The mean operating time was longer in the TOETVA group (102.9 ± 26.1 mins) than that in the open surgery group (66.8 ± 23.8 mins) with *p* = 0.0001. Cosmetic outcomes and overall satisfaction were significantly greater in the TOETVA group *p* = 0.0001. The SF-36 QOL scores of the patients in the TOETVA group were generally higher than the open surgery group.

**Conclusions:**

TOETVA has been widely used with a low complication rate, cosmetic appeal, and surgical efficacy. Postoperative quality of life, cosmetic outcomes, and overall satisfaction were significantly superior to the open surgery group.

## 1. Introduction

In recent years, thyroid nodules have increased worldwide, with the incidence of thyroid nodules detected using ultrasound accounting for 68% of the general population [1]. Of this number, well-differentiated thyroid cancer made up 5–10% [2]. According to GLOBALCAN 2020, thyroid cancer ranks 9th in both genders with 586.202 new cases diagnosed in the world [3]. The proportion of well-differentiated thyroid cancers with two main histological types (papillary and follicular) was greater than 90% of thyroid cancers. Well-differentiated thyroid cancer is considered to have an excellent prognosis with 20-year overall survival [2, 4].

Surgery is the cornerstone of therapy for patients suffering from both thyroid nodules and thyroid cancers [2, 5, 6]. Over the past 20 years, several remote access approaches have been used in the area of thyroidectomy. The transoral endoscopic thyroidectomy vestibular approach (TOETVA) has recently been developed widely worldwide with a low complication rate, cosmetic appeal, and surgical efficacy [7–9]. This remote procedure is indicated for both benign thyroid nodules and thyroid cancers. Although it is proven that TOETVA is safe, feasible, and has invisible scarring, it is still an experimental technique with less data available regarding the patient's quality of life after surgery. At our center, Hanoi Medical University Hospital, TOETVA has been developed and applied since 2018, and it has proven the superior efficacy and patient satisfaction in comparison with other methods such as auxillary endoscopic thyroidectomy and open surgery [10–13].

The incidence rate of thyroid tumors is recently increasing in people, especially in young females, with long life expectancy. Therefore, in addition to the treatment outcome, the quality of life of the patients also needs to be researched. In the world, several studies have been conducted on the quality of life of patients following thyroid surgery. Although well-differentiated thyroid cancer has an excellent prognosis, several studies have found that patients who have undergone thyroid surgery have a worse quality of life than the general population [14–17]. However, our knowledge about the quality of life of patients after thyroid surgery by TOETVA is based on very limited data due to the novelty of the technique and the short duration of follow-up time. The study by Kasemsiri et al. demonstrated that patients in the endoscopic oral vestibular thyroidectomy groups have better aesthetic outcome and quality of life than in the open surgery group. Kasemsiri's study was one of the first research studies in the world to evaluate the quality of life of patients after thyroid surgery by TOETVA but with a small sample size in the group of patients with benign thyroid nodules [17]. Therefore, the purpose of our study was to investigate postoperative health-related quality of life in patients with benign thyroid nodules and differentiated thyroid cancer undergoing TOETVA and open surgery using the SF-36 quality of life scale.

## 2. Materials and Methods

### 2.1. Study Design

The study was designed as a prospective study, in which 121 patients undergoing thyroid surgery were split into two groups: 60 patients in the TOETVA group and 61 patients in the open surgery group. Patients choose their surgical procedures according to their preferred option after being fully explained on the advantages and disadvantages of each type of surgical treatment. The data were collected from a single center, the Department of Oncology and Palliative Care at the Hanoi Medical University Hospital, from May 2020 to November 2021. All operations were performed by one team. The ethical endorsement of the study was given by the Ethical Council of the Hanoi Medical University.

### 2.2. Eligible Participants

**Figure 1:**
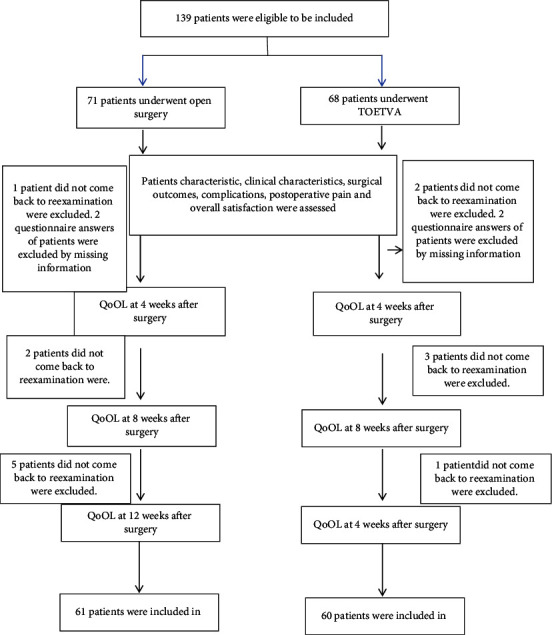
The flow chart of the follow-up process.

Eligible participants in both groups were 18–79 year olds with benign thyroid nodules less than 6 cm in diameter or well-differentiated thyroid cancer less than 2 cm in diameter without lateral lymphadenopathy metastases. All patients were explained the study protocol and agreed to participate in the research. Patients have ability to complete questionnaires in Vietnamese and answer the interviews. Patients filled two questionnaires in the paper after being fully explained the method of filling in the questionnaires and the meaning of all the categories in the questionnaires. Patients were excluded if the answers to questionnaire were not informative or patients did not feel comfortable to fill in at any time of the research period. Patients with total thyroidectomy, lobectomy, and isthmectomy, including central lymph node dissection, were eligible to be included. Patients were excluded if they had thyroid cancer suspected of lateral lymphadenopathy metastases, extended invasion to surrounding tissue (≥T4a), or distant metastases after a thorough evaluation, including ultrasound, fine needle aspiration, ultrasound, CT, MRI, or intraoperative suspicion. Patients were also excluded if they had a history of prior neck surgery, Grave's disease, toxic multinodular goiters, extended thyroidectomy to surrounding tissue, large substernal goiter, coagulopathy, or those who had been through the technique converted from endoscopic to open approach during surgery. Patients were excluded if they did not agree in participating in the research or lost to follow-up at any time of the research period. The technique of the TOETVA procedure was described in our previous studies [10, 11, 13]. The flow chart of follow-up process was presented in Figure 1.

## 3. Materials

The two groups were compared in terms of demographic characteristics (the patient's gender, age, education, employment status, and marital status), clinical characteristics (including tumor size, pathology, type of surgery, and lymph node dissection), surgical outcomes (operation time, intraoperative blood loss, the VAS score, length of hospitalization, and satisfaction), and complications (seroma, hematoma, hypoparathyroidism, hoarseness, and wound infection). We did not regularly use the intraoperative neuro-monitoring system in both groups because intraoperative neuro-monitoring is more necessary in thyroid reoperations to reduce risk of recurrent laryngeal nerve (RLN) injury [18]. Postoperative pain was evaluated by using the Visual Analogue Scale (VAS) from 0 (no pain) to 10 (worst pain imaginable) on postoperative days 1 and 2. Overall satisfaction after surgery ranged from 0 to 5, and cosmetic outcome scores ranged from 5 to 0.

The patients have been followed-up including surgical outcomes, cosmetic satisfaction, and quality of life. These criteria were accessed at 4 weeks, 8 weeks, and 12 weeks after the surgery using SF-36 and thyroid surgery-specific questionnaire. Items of thyroid surgery-specific questionnaire were accessed at 4 weeks, including voice impairment, neck movement, shoulder movement, swallowing impairment (range from 0 to 4 : 0, never; 1, almost never; 2, sometimes; 3, almost always; and 4, always); tingling sensations and numbness in the neck and chin areas (0, no pain or other abnormal sensation; 1, minimum; 2, moderate; and 3, severe); physical activity and psychosocial impairment (0, no damage; 1, almost never (occasionally); 2, sometimes; 3, almost always; and 4, always). In total thyroidectomy patients with coexistent bilateral thyroid disease or without central lymphadenopathy metastases, patients were tested for thyroglobulin (Tg) and antiTg, and we performed thyroid ultrasound at each time of reexamination at 4 weeks, 8 weeks, and 12 weeks.

SF-36 has been widely used in measuring HRQoL in thyroid cancer in previous studies in different languages [19]. There are 36 questions and categorized into an 8-parameter profile of scores: physical functioning (PF; 10 items), general health (GH; 5 items), role-physical (i.e., role limitations due to the physical health problems, RP; 4 items), bodily pain (BP; 2 items), social functioning (SF; 2 items), vitality (VT; 4 items), role-emotional (i.e., role limitations due to emotional problems, RE; 3 items), and mental health (MH; 5 items) [20]. For each parameter, the higher score (ranging from 0 to 100) indicated the better health quality of life.

### 3.1. Data Management and Analysis

Data were input by Microsoft Excel 2016. All the data analysis was performed by SPSS 19.0.1 (IBM Corporation, Armonk, New York, USA) and GraphPad Prism 8.4.2. All continuous variables were expressed as the mean ± SD. The normalcy of the data distribution was verified by the Kolmogorov–Smirnov test. All the domains of the SF-36 questionnaire were determined by the Mann–Whitney *U* test. The categorical variable was tested using the chi-square test, while the small cell variables were compared using the exact Fisher test. The statistical significance of data was defined by *p* value less than 0.05.

## 4. Results

From May 2020 to November 2021, a total of 121 patients who met all the eligible criteria were assigned to the TOETVA group or open surgery group. The basic and clinico-pathological demographic characteristics of two groups were compared in Table 1. In general, gender and socio-economic status, including education, employment status, and marital status, were similar in two groups (*p* > 0.05), except for age. Patients in the TOETVA group are significantly younger than patients in the open surgery group (35.8 ± 10.3 vs 46.9 ± 11.5, *p* < 0.001). In terms of clinical characteristics, participants in both groups presented with similar features in ultrasound characteristics, postoperative pathology, and type of thyroid surgery (*p* > 0.05). The proportions of well-differentiated thyroid cancer pathology in the TOETVA group and open surgery group were 76.7% and 72.1%, respectively (*p*=0.9). There was 1 patient (1.07%) in the open surgery group who had the pathology results NIFTP (noninvasive follicular thyroid neoplasm with papillary-like nuclear features).

The mean operating time was longer in the TOETVA group 102.9 ± 26.1 mins than that in the open surgery group 66.8 ± 23.8 mins with *p*=0.0001. The postoperative pain scores were similar between two groups at POD 1 (*p*=0.637) and at POD 2 (*p*=0.14). The average length of stay in the hospital in the TOETVA group (5.9 ± 1.1 days) was significantly shorter than in the open surgery group (6.6 ± 2.0 days) with *p*=0.034 (Table 2).

Among 60 patients who underwent TOETVA surgery, 7 patients experienced postoperative hoarseness, while the figure for open surgery groups was 9 patients (*p*=0.62). All patients recovered within 4 weeks after surgery. One patient had complications of bleeding after the operation in the open surgical group but did not require reoperation due to excessive bleeding. There were no complications of postsurgical bleeding in the TOETVA groups (Table 3).

### 4.1. Thyroid Surgery-Specific Questionnaire

The TOETVA group performed better than the open surgery group in items of thyroid surgery-specific questionnaire but no statistical significance. Cosmetic outcomes and overall satisfaction were significantly better in the TOETVA group at 4 weeks after surgery (*p*=0.0001 and *p*=0.002, respectively) (Table 4).

### 4.2. SF-36 Questionnaire Scores

**Figure 2:**
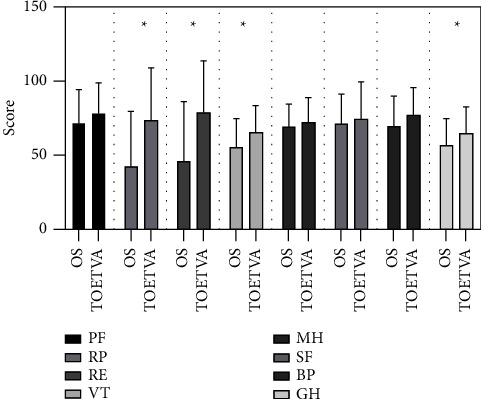
SF-36 score comparison between the TOETVA group and open surgery group at 4 weeks after surgery. BP = bodily pain, GH = general health, GP = general population, MH = mental health, RE = role-emotional, RP = role-physical, SF = social functioning, and VT = vitality. ^*∗*^ = statistical significance.

The SF-36 QOL scores of the patients in the TOETVA group were generally better than in the open surgery group in all parameters (Figure 2). The RP, RE, VT, BP, and GH scores of patients in the TOETVA group were statically significantly higher than in the open surgery group at 4 weeks after surgery. The results suggested that the physical wellbeing of patients in the TOETVA group was better than in the surgery group. At 8 weeks after operation, RE scores of patients in the TOETVA group were better than in the open surgery group. The SF-36 score of patients was not significant between two groups at 12 weeks (Table 5).

## 5. Discussion

Oral vestibular endoscopic thyroidectomy is a new technique and promising approach as it is both minimally invasive and optimally cosmetic. A number of studies have been proven the exact role of this method with a low complication rate, cosmetic appeal, and surgical efficacy [7–9]. Because of long life expectancy and a major proportion of young female patients with thyroid nodules, cosmetic satisfaction and postoperative QoL are increasingly concerned besides surgical outcomes. Our study determined the surgical outcome and quality of life of 121 patients newly diagnosed with thyroid tumors who underwent either TOETVA or open thyroidectomy. There were no statistically significant differences between the two groups in sex, age, eco-social status, tumor size, ultrasound characteristics, postoperative pathology, and the type of thyroid surgery. However, the mean operating time was significantly longer in the TOETVA group than that in the open surgery group. This may be the consequence of the aid of an unskilled operative assistant. In addition, because we performed prophylactic central lymph node dissection for all patients diagnosed with thyroid cancer, it took longer time to complete the surgery. Although the longer operation time was one of the drawbacks of this technique, it can be mitigated with improving surgical experience [21–23]. The average length of stay in the hospital in the TOETVA group (5.9 ± 1.1 days) was significantly shorter than in the open surgery group (6.6 ± 2.0 days) with *p*=0.034. Hospital stay was calculated from when patients were admitted to the hospital until they were discharged. During the COVID 19 pandemic, all of our patients had to admit 2 days before surgery for the PCR test and prepare for the operation. In addition, unlike in Western countries, Vietnamese patients wish to stay in the hospital until they recover completely.

SF-36 has been widely used in measuring HRQoL in thyroid cancer in previous studies [19]. However, SF-36 cannot evaluate thyroid cancer-specific symptoms which can impact the quality of life. Thus, the questionnaire of symptoms related to the thyroid surgery questionnaire was included in our study to evaluate important aspects regarding thyroid-specific symptoms after surgery [24]. According to Gou, quality of life of patients with PTC was worst at 1 month after thyroid surgery and recovered within 6 months after surgery but did not reach those of the general population level [25]. The cause may be due to transient symptoms after surgery such as numbness, tingling, voice impairment, swallowing impairment, and neck movement impairment which can be recovered [26–28]. In our present study, these symptoms were generally better in the TOETVA group than the open surgery group, but there was no statistically significantly difference. Most of these symptoms were recovered within 3 months after the surgery in our patients.

Our study compared the HRQoL of patients with thyroid nodules with different treatment strategies: TOETVA and open surgery at 4 weeks, 8 weeks, and 12 weeks after operation. Significant differences in many parameters of the SF-36 questionnaires were showed. The open surgery group reported more problems associated with RP, RE, and GH than the TOETVA group. GH, RP, and RE represent the general health of the patients and the physical and emotional restrictions on daily activities or work, respectively. These results are consistent with the study by Kasemsimi et al. [17] reported in 2020. The explanation for this may be surgical trauma or complications [25, 29, 30]. In our study, no patients in the TOETVA group reported significant complications. Given there were numerous patients with thyroid cancer in our study, another explanation is that patients with thyroid cancer are constantly concerned about recurrence and metastasis during long-term follow-up [31]. According to Lubitz et al., the parameter of RE remained declining years after surgery even without the evidence of recurrence of cancer [15]. Hedman et al. also showed that 48% of patients with thyroid cancer worried about recurrence, while in fact, only 7% of them actually had disease recurrence [32].

Cosmetic outcome and overall satisfaction were significantly better in the TOETVA group in comparison with open surgery group in our research. There were numerous studies which reported that during the first year after the surgery, the problem of scarring was one of the major reasons of decrease in the quality of life [28]. This was one of the major causes of decline in the quality of life in the surgery group. The problem of permanent scarring was more common in patients who underwent surgery than those who underwent TOETVA. Because most patients were women and prognosis of the thyroid diseases is good with long life expectancy, apparent scar may have negatively impacted the patients in their daily life in many aspects from communication, fashion, and even career development [33, 34]. In addition, many patients obsessed the obvious scar on their body regardless of the scar type, which had negatively impact on confidence and self-esteem and quality of life [32–35]. Therefore, remote approach thyroid surgery without a visible scar would improve quality of life of patients in the long term. The development of our research may have health implication for thyroid cancer patients in the future.

We acknowledge that this study has some limitations. First, although the differences we observed were statistically significant, the number of patients included in our study was limited, and the results may not be clinically meaningful. In addition, the follow-up time is only 12 weeks, which may not determine the surgical outcome and quality of life in the long term. Short period of following may overestimate the drawbacks of surgery on quality of life of patients due to surgical trauma or complication. Finally, preoperative quality of life was unknown in both groups because patients were shocked after diagnosis and tended to refuse to answer the questionnaire. Further studies should be conducted with large samples, fully preoperative assessment, and longer follow-up time.

## 6. Conclusion

TOETVA has been widely used with a low complication rate, cosmetic appeal, and surgical efficacy. Postoperative quality of life, cosmetic outcomes, and overall satisfaction were significantly better than the open surgery group.

## Figures and Tables

**Table 1:** Patient characteristics.

	TOETVA (*n* = 60)	Open (*n* = 61)	*p* value
Age (years)	35.8 + 10.3	46.9 + 11.5	<0.001

Gender			
Female	54 (90)	54 (88.5)	0.793
Male	6 (10)	7 (11.5)	

Education			
>high school graduate	57 (95)	54 (88.5)	0.78
<high school graduate	3 (5)	7 (11.5)	

Employment status			
Employed	46 (76.7)	43 (70.5)	0.76
Unemployed	14 (23.3)	18 (29.5)	

Marital status			
Married	48 (80)	52 (85.2)	0.81
Nonmarried	12 (20)	9 (14.7)	

Thyroid size (mm)	10.9 ± 9.0	11.9 ± 9.1	0.56

Pathology, *n* (%)			
Benign goiter	14 (23.3)	16 (26.2)	0.9
Thyroid cancer	46 (76.7)	44 (72.1)	
Others	0 (0)	1 (1.07)	

**Table 2:** Surgical outcomes.

	TOETVA (*n* = 60)	Open (*n* = 61)	*p* value
Types of thyroid surgery, *n* (%)			
Total thyroidectomy	14 (23.3)	16 (62.5)	0.637
Hemithyroidectomy and isthmus	39 (65.0)	36 (59.0)	
Hemithyroidectomy	7 (11.6)	9 (14.8)	

Lymph node dissection			
No	10 (16.7)	11 (18.0)	0.634
Unilateral	39 (65)	36 (59.0)	
Bilateral	11 (18.3)	14 (23.0)	

Operative time (minutes)	102.9 ± 26.1	66.8 ± 23.8	0.0001

Blood loss (ml)	5.6 ± 2.2	6.6 ± 2.7	0.127

VAS score			
Postoperative day 1	3.76 ± 0.19	3.8 ± 0.9	0.637
Postoperative day 2	2.29 ± 0.83	2.57 ± 0.91	0.14
Hospital stays (days)	5.9 ± 1.1	6.6 ± 2.0	0.034

Values are presented as the mean ± standard deviation; LND: lymph nodes dissection.

**Table 3:** Perioperative complications.

Complications	TOETVA (*n* = 60)	OS (*n* = 61)	*p* value
Seroma	1 (1.7)	2 (3.3)	0.51
Hematoma	0	1 (1.6)	0.55
Hypoparathyroidism	0	0	—
Hoarseness	7 (11.7)	9 (14.8)	0.62
Postoperative bleeding	0	0	—
Wound infection	0	0	—

Values are presented as the mean ± standard deviation; LND, lymph nodes dissection.

**Table 4:** Comparison symptoms-related thyroid surgery between the TOETVA group and open surgery group at 4 weeks after surgery.

Complications	TOETVA (*n* = 60)	OS (*n* = 61)	*p* value
Numbness	1.2 ± 1.1	1.6 ± 1.3	0.078
Tingling	0.4 ± 0.6	0.6 ± 0.7	0.07
Cosmetic	4.6 ± 0.5	4.1 ± 0.9	0.0001
Voice impairment	1.2 ± 1.1	1.6 ± 1.3	0.08
Swallowing impairment	1.18 ± 0.9	1.1 ± 1.0	0.09
Neck movement impairment	0.8 ± 0.8	0.7 ± 0.8	0.47
Sloulder movement impairment	0.6 ± 0.5	0.6 ± 0.8	0.771
Physical activity reduction	0.9 ± 0.9	1.1 ± 1.0	0.273
Psychosocial impairment	0.5 ± 0.8	0.7 ± 0.8	0.69
VAS	1.2 ± 1.3	1.4 ± 1.5	0.443
Overall satisfaction	4.6 ± 0.5	4.2 ± 0.9	0.002

**Table 5:** SF-36 score comparison between the TOETVA group and open surgery group at 4 weeks, 8 weeks, and 12 weeks after surgery.

Time	4 weeks			8 weeks			12 weeks		
SF36	TOETVA	OS	*p* value	TOETVA	OS	*p* value	TOETVA	OS	*p* value
Physical function (PF)	78.5 ± 20.7	71.5 ± 23.1	0.09	86.3 ± 17.9	85.0 ± 18.4	0.69	91.3 ± 15.9	88.2 ± 14.7	0.26
Role physic (RP)	74.6 ± 34.9	42.6 ± 37.2	0.0001	86.6 ± 23.8	81.5 ± 29.0	0.29	94.7 ± 13.2	89.3 ± 20.2	0.08
Role emotion (RE)	79.3 ± 34.7	46.4 ± 40.0	0.0001	84.3 ± 22.2	67.3 ± 25.6	0.0002	85.7 ± 18.9	78.6 ± 24.7	0.08
Vitality (VT)	66.1 ± 17.6	55.7 ± 19.1	0.003	76.8 ± 22.3	70.1 ± 26.7	0.14	78.7 ± 20.5	74.6 ± 19.2	0.26
Mental health (MH)	72.8 ± 16.5	69.9 ± 14.6	0.32	83.7 ± 20.3	81.5 ± 21.8	*P*=0.56	89.0 ± 16.9	87.8 ± 10.9	0.64
Social function (SF)	75.2 ± 24.4	71.6 ± 20.0	0.37	84.8 ± 13.2	81.2 ± 17.3	*P*=0.20	88.2 ± 15.7	83.4 ± 19.5	0.14
Bodily pain (BP)	77.5 ± 18.3	70.3 ± 19.8	0.043	81.1 ± 19.7	79.7 ± 23.1	0.72	92.1 ± 19.7	89.2 ± 19.9	0.42
General health (GH)	65.4 ± 17.5	56.6 ± 18.1	0.009	73.1 ± 22.1	71.4 ± 25.3	0.69	78.0 ± 20.1	76.5 ± 15.6	0.65

## Data Availability

All the data underlying the results are available as part of the article, and no additional source data are required. All data sources described in this study are directed at the corresponding author.

## ABBREVIATIONS

BP: Bodily pain
GH: General health
GP: General population
HRQoL: Health-related quality of life
QoL: Quality of life
MH: Mental health
PTC: Papillary thyroid carcinoma
RE: Role-emotional
RP: Role-physical
SF: Social functioning
VT: Vitality.
